# Development and Validation of Prediction Models for Severe Complications After Acute Ischemic Stroke: A Study Based on the Stroke Registry of Northwestern Germany

**DOI:** 10.1161/JAHA.121.023175

**Published:** 2022-03-05

**Authors:** Anna K. Bonkhoff, Nicole Rübsamen, Christian Grefkes, Natalia S. Rost, Klaus Berger, André Karch

**Affiliations:** ^1^ J. Philip Kistler Stroke Research Center Massachusetts General Hospital Harvard Medical School Boston MA; ^2^ Institute of Epidemiology and Social Medicine University of Muenster Albert‐Schweitzer‐Campus 1 Muenster Germany; ^3^ Cognitive Neuroscience Institute of Neuroscience and Medicine Research Centre Juelich Juelich Germany; ^4^ Department of Neurology Department of Neurology University Hospital Cologne and Medical Faculty University of Cologne Germany

**Keywords:** ischemic stroke, machine learning, mortality, prediction, severe outcomes, Mortality/Survival, Quality and Outcomes, Ischemic Stroke

## Abstract

**Background:**

The treatment of stroke has been undergoing rapid changes. As treatment options progress, prediction of those under risk for complications becomes more important. Available models have, however, frequently been built based on data no longer representative of today’s care, in particular with respect to acute stroke management. Our aim was to build and validate prediction models for 4 clinically important, severe outcomes after stroke.

**Methods and Results:**

We used German registry data from 152 710 patients with acute ischemic stroke obtained in 2016 (development) and 2017 (validation). We took into account potential predictors that were available at admission and focused on in‐hospital mortality, intracranial mass effect, secondary intracerebral hemorrhage, and deep vein thrombosis as outcomes. Validation cohort prediction and calibration performances were assessed using the following 4 statistical approaches: logistic regression with backward selection, *l*1‐regularized logistic regression, k‐nearest neighbor, and gradient boosting classifier. In‐hospital mortality and intracranial mass effects could be predicted with high accuracy (both areas under the curve, 0.90 [95% CI, 0.90–0.90]), whereas the areas under the curve for intracerebral hemorrhage (0.80 [95% CI, 0.80–0.80]) and deep vein thrombosis (0.73 [95% CI, 0.73–0.73]) were considerably lower. Stroke severity was the overall most important predictor. Models based on gradient boosting achieved better performances than those based on logistic regression for all outcomes. However, area under the curve estimates differed by a maximum of 0.02.

**Conclusions:**

We validated prediction models for 4 severe outcomes after acute ischemic stroke based on routinely collected, recent clinical data. Model performance was superior to previously proposed approaches. These predictions may help to identify patients at risk early after stroke and thus facilitate an individualized level of care.

**Nonstandard Abbreviations and Acronyms**
NIHSS: National Institutes of Health Stroke Scale

**Clinical Perspective**

**What Is New?**

- The present study is the first to rely on a large stroke registry comprising recent clinical data on 152 710 patients with acute ischemic stroke and test the performances of several machine‐learning algorithms in their prediction of 4 severe complications (in‐hospital mortality, intracranial mass effects, secondary intracerebral hemorrhage, and deep vein thrombosis) after stroke.
- In‐hospital mortality and intracranial mass effects in particular could be predicted with high accuracies and areas under the curve of ≈0.90, with admission stroke severity being the most important predictor.
- Gradient boosting–based models were shown to consistently outperform logistic regression; however, differences in areas under the curve were small.

**What Are the Clinical Implications?**

- Severe complications after stroke can be accurately predicted by employing routinely collected clinical data, as exemplarily recorded in stroke registries.
- Patients at risk for certain complications may be identified early after stroke by the model predictions presented here and receive an individualized level of care, for example, featuring specific additional diagnostic tests, such as venous Doppler of the legs.
- Validated models are openly available for the generation of predictions for new patients: https://github.com/AnnaBonkhoff/Predict_severe_complications_after_stroke

Stroke mortality has decreased globally in recent years.^1^ Nonetheless, severe complications after stroke continue to reduce the chance of a good functional outcome, as they may delay and impede recovery after stroke.^2^ Many research studies have focused on identifying those at highest risk for severe complications after stroke in an aim to offer tailored therapies for individual patients with stroke and hence optimize clinical workflows.^3^ These efforts have focused on the prediction of early mortality, intracranial mass effect, and secondary intracerebral hemorrhage as well as deep vein thrombosis (DVT; eg, in the lower extremity) and provided promising results.^4^, ^5^, ^6^, ^7^ However, many of these currently available prediction models have been developed on stroke data gathered years ago or were trained using data from a larger time span to increase sample sizes. This circumstance may hamper a smooth implementation of the models in current clinical routine given that stroke care has changed considerably during the past 2 decades. These changes comprise the widespread use of intravenous thrombolysis and mechanical thrombectomy, as well as the extension of time windows after symptom onset when these acute therapies can be administered, to name only a few examples.^8^, ^9^ Conceivably, all of these changes have a substantial influence on the occurrence of complications and their prediction.

We used recent data from a large German stroke registry^10^, ^11^ to predict intrahospital mortality, intracranial mass effect, secondary intracerebral hemorrhage, and DVT after ischemic stroke. To this end, we developed and validated 4 individual prediction models. In addition, we systematically evaluated if the use of advanced model‐building strategies might improve the prediction accuracy.

## Methods

### Data and Code Availability

Data analysis was conducted using jupyter notebooks in a python3.7 environment, particularly using implementations offered through the package scikit‐learn.^12^

The code and trained models to generate outcome predictions for new patient data and a corresponding step‐by‐step documentation is openly available online: https://github.com/AnnaBonkhoff/Predict_severe_complications_after_stroke. The authors agree to make the data available to any researcher for the express purposes of reproducing the results presented here based on a written data transfer agreement and with the explicit permission for data sharing by the local institutional review board. The original data collection tool can be accessed at https://www.medizin.uni‐muenster.de/qsnwd/downloads.html (“Spezifikationen”), whereas a version translated into English can be found in Data S1.

### Study Population

Data on patients with stroke originated from the Stroke Registry of Northwestern Germany. This registry has prospectively collected demographic and clinical characteristics of adult patients with stroke since the year 2000, consisted of a network of 155 hospitals at the time of data collection for this study, and has been described in detail previously.^10^, ^11^ Hospital participation in the stroke registry project is voluntary. Participation is, however, a mandatory prerequisite for stroke unit certification through the German Stroke Society, which serves as motivating factor. In this study, we included data on any patient hospitalized in 2016 or 2017 with ischemic stroke and the *International* *Classification of Diseases, Tenth Revision* (*ICD‐10*) diagnosis code I63. Data from 2016 were used for model development, and data from 2017 were used for model validation. Registry data consisted of routine clinical stroke data, such as the National Institutes of Health Stroke Scale (NIHSS)–defined stroke severity, that were obtained by experienced physicians. Data were anonymized, centrally quality controlled, and stored at the coordinating center at the University of Muenster, Germany. The ethics committee of the Westphalian Board of Physicians and the University of Muenster approved the study design. Because the identity of each documented patient is completely anonymized at the point of data collection, no study‐specific informed consent was obtained. This study complies with the Transparent Reporting of a Multivariable Prediction Model for Individual Prognosis or Diagnosis reporting guideline.^13^

### Predictor Variables and Outcomes

The aim of this study was to develop practically applicable prediction models for severe complications after stroke in a broad, unselected sample of patients with acute ischemic stroke. We considered every variable of the standardized registry data set as potential predictor if it was continuously acquired in both 2016 and 2017 and was recorded within the first 24 hours after admission. This latter limitation was included to ensure applicability of the prediction model as early as possible. We focused on the prediction of severe adverse outcomes after stroke: early mortality (within the first 7 days after admission), intracranial mass effect, secondary intracerebral hemorrhage, and DVT, for example, of veins in the lower extremity. In the case of secondary intracerebral hemorrhage, we performed a subgroup analysis after the exclusion of all patients receiving intravenous thrombolysis. All events were recorded as present versus not present (and not in any graded way). Diagnoses themselves were passed on by each participating hospital and relied on their clinical pathways. For example, a patient might have been diagnosed as having an intracranial mass effect noninvasively based on classic neuroimaging findings (eg, compression of ventricles and midline shift) in combination with clinical symptoms (eg, reduced level of consciousness). We conducted analyses on a complete case basis, that is, we excluded patients with missing data for any of our considered variables (excluded patients, 4.3% overall; cf. Data S2). We compared the key covariates age, sex, and Rankin Scale upon admission between included and excluded patients to assess potential selection biases. A full list of all included 47 predictor variables and outcomes is presented in Table 1 and Table S1. Exact numbers of included and excluded patients and data of key comparisons are presented in Tables S2 through S4.

**Table 1:** Stroke Sample Characteristics

	2016 and 2017, N=146 062
Age, y	72.7 (13.1)
Female sex	69 234 (47.4)
Situation of living, before stroke	
Independently in own home	117 055 (80.1)
Care at home	15 847 (10.9)
Nursing home	13 160 (9.0)
Comorbidities	
Diabetes	42 944 (29.4)
Hypertension	124 754 (85.4)
Previous myocardial infarct	14 246 (9.8)
Previous stroke	38 089 (26.1)
Hypercholesterinaemia	84 644 (58.0)
Atrial fibrillation	
Yes, known before stroke	28 962 (19.8)
Yes, previously unknown	13 455 (9.2)
Stroke severity and symptoms at admission	
Stroke severity (NIHSS)	5.9 (6.2)
	4 (6)
Motor impairments	95 636 (65.5)
Language impairments	44 684 (30.6)
Speech impairments	63 962 (43.8)
Swallowing impairments	32 168 (22.0)
Consciousness	
Awake	134 357 (92.0)
Soporific‐stuporous	10 034 (6.9)
Comatose	1671 (0.01)
Rankin scale	
0	7749 (5.3)
1	20 541 (14.1)
2	35 672 (24.4)
3	34 920 (23.9)
4	23 839 (16.3)
5	23 341 (16.0)
Median (interquartile range)	3 (2)
Barthel index: bladder function	
0	29 459 (20.2)
5	18 893 (12.9)
10	97 710 (66.9)
Median (interquartile range)	10 (5)
Barthel index: transfer	
0	27 596 (18.9)
5	24 527 (16.8)
10	35 666 (24.4)
15	58 273 (39.9)
Median (interquartile range)	10 (10)
Barthel index: mobility	
0	34 310 (23.5)
5	27 645 (18.9)
10	36 117 (24.7)
15	47 990 (32.9)
Median (interquartile range)	10 (10)
Admission, times, and therapies	
Intravenous thrombolysis	24 989 (17.1)
Intraarterial thrombectomy and thrombolysis	10 706 (7.3)
Time from symptom onset until admission	
<1 h	11 825 (8.1)
1–2 h	23 177 (15.9)
2–3 h	13 889 (9.5)
3–3.5 h	4697 (3.2)
3.5–4 h	4229 (2.9)
4–6 h	13 366 (9.2)
6–24 h	29 721 (20.4)
24–48 h	10 923 (7.5)
>48 h	17 539 (12.0)
Imaging before admission	15 270 (10.5)
Intensive care admission	7752 (5.3)
Stroke characteristics	
TOAST classification	
Atherothrombotic	33 314 (22.8)
Embolic	46 097 (31.6)
Microangiopathic	30 003 (20.5)
Competing	5754 (3.9)
Other	5153 (3.5)
Uncertain	25 741 (17.6)
Large vessel stenosis	
Stenosis	137 411 (94.1)
No stenosis	5335 (3.7)
Unknown, no diagnostic tests	3316 (2.3)
Complications	
In‐hospital mortality	7683 (5.3)
Intracranial mass effect	2411 (1.7)
Secondary intracerebral hemorrhage	2580 (1.8)
Deep vein thrombosis	606 (0.4)

Please note that the variable “Admission to intensive care” was only included in the prediction models of early mortality. Although admission to intensive care necessarily occurs before a fatal outcome, the temporal order was not known for any of the other 3 complications (ie, we could not exclude that admission to intensive care was a consequence of a complication). Continuous variables are presented as mean (SD) and categorical variables as absolute count (percentage). NIHSS indicates National Institutes of Health Stroke Scale. TOAST stands for the Trial of Org 10172 in Acute Stroke Treatment.

### Model Development

We developed 4 different models to predict each of the adverse outcomes after stroke. The classic model relied on logistic regression in combination with a backward stepwise procedure. In this scenario, we started with the full model considering any available input variable to predict the specific outcome. The variable associated with the highest *P* value was then dropped from the model if it additionally exceeded a threshold of *P*>0.01. The variable selection process stopped once all remaining variables were significantly associated with the outcome (level of significance, *P*<0.01). In addition, we employed 3 modern model‐building strategies representing different types of learning algorithms typically applied in the field of machine learning. This includes a *l_1_*‐regularized regression model^14^; a k‐nearest neighbor classifier^15^; and a tree‐based model, the gradient boosting classifier.^16^

During model development, we first performed a downsampling step. There were substantially fewer patients presenting with a specific complication than patients without this complication. For example, only 1.8% of all patients experienced an intracerebral hemorrhage, while 98.2% did not. Hence, we randomly selected (without replacement) a subset of the larger, nonaffected patient group to establish a group balance (cf. Data S3 for further motivations). By these means, we developed models in samples of 50% affected and 50% nonaffected patients. This downsampling step was repeated 100 times. The subsequent internal validation scheme depended on the respective prediction model. In case of logistic regression, we initiated a 4:1 train:test set split after each downsampling step. The backward stepwise selection of input variables and model fitting was performed in the train data set, whereas the prediction performance was then obtained for the hold‐out test set. The 3 other approaches, the *l*1‐regularized logistic regression, k‐nearest neighbor, and gradient boosting classifier, entered a nested cross‐validation to securely run a hyperparameter optimization step intended to maximize prediction performance.^17^ After an initial 4:1 train:test set split, we conducted a grid search to find the best hyperparameter settings via 5‐fold cross‐validation in the train set (cf. Table S5 for details on hyperparameter choices). The best performing model within this inner loop was then tested in the hold‐out test set to get an estimate of an unbiased internal validation prediction performance. We measured out‐of‐sample prediction performance as area under the receiver operating curve (AUC) in the test sets. This measure therefore took into account the true positive and false positive rates at decision thresholds varying from 0 to 1. Lastly, we recorded in how many of the 100 downsampling repetitions a variable was chosen to stay in the model in the case of backward stepwise regression. In the case of the gradient boosting classifier, we noted the average feature importance. This importance can be explicitly computed for each input variable, allows their ranking and comparison (cf. Data S3 for details). We present odds ratios for the most stably selected input variables of logistic regression models as well as group averages (ie, patients with a specific outcome versus those without) for the most relevant input variables to allow for conclusions on the likely directionality of effects.

### Validation in Time

We implemented a temporal validation by testing the developed models on registry data obtained in the subsequent year from January 1, 2017, to December 31, 2017. Included input and output variables remained the same. Although we had inserted a downsampling step during model development and had thus considered the same number of patients with and without a certain complication, we considered the entire sample without downsampling for model validation. We calibrated the models developed in the 2016 data set by weighing the model’s probabilistic predictions according to the fractions of patients with severe outcomes in the development and validation cohort. The final models were then employed to obtain a nonoptimistic estimate of the AUC. In addition, we visually evaluated the calibration of our prediction models in calibration plots and obtained Brier scores.^18^

## Results

Overall, we considered trajectories of 152 710 patients with ischemic stroke included in the Stroke Registry of Northwestern Germany. Patients admitted to a participating hospital from January 1, 2016, to December 31, 2016, with complete data contributed to the development cohort (N=74 749 of 76 019, 98.3%), whereas patients of the subsequent year were assigned to the validation cohort (N=71 313 of 76 691, 93.0%; cf. Tables S2 through S4 for comparisons of included and excluded patients). The mean age of all patients was 72.7 years (SD, 13.1 years), 47.4% were women, median stroke severity at admission was determined as an NIHSS score of 4 (interquartile range [IQR], 6). Further baseline characteristics are summarized in Table 1.

Calculated during the 2 years of data recording, complication rates were 1.7% for intracranial mass effect (n=2411), 1.8% for intracerebral hemorrhage (n=2580), and 0.4% for DVT (n=606). Mortality within the first week after admission was 5.3% (n=7683).

### Prediction Results in the Validation Data Set (2017)

After developing the 4 competing models in the 2016 data set, we observed best 2017 validation data set prediction performances for the outcomes early mortality and intracranial mass effect with AUC values of 0.90 (95% CI, 0.90–0.90). Validation data set prediction performances for secondary intracerebral hemorrhage and DVT were 0.80 (95% CI, 0.80–0.80) and 0.73 (95% CI, 0.72–0.73), respectively (Table 2). Validation data set prediction performance for intracerebral hemorrhage remained almost the same when restricting analyses to patients who did not receive any thrombolytic therapy (AUC, 0.79 [95% CI, 0.79–0.79]; Table S6). The gradient boosting classifier achieved best results for all 4 outcomes and outperformed logistic regression in all cases. However, prediction performances across classifiers were generally comparable and within a very narrow range of AUCs (largest difference: DVT, 0.73 [95% CI, 0.72–0.73] versus 0.71 [95% CI, 0.71–0.71] gradient boosting classifier versus logistic regression).

**Table 2:** Prediction Results for All 4 Outcomes and Prediction Models in the Temporal Validation Cohort

Classifier	In‐hospital mortality	Intracranial mass effect	Secondary intracerebral hemorrhage	Deep vein thrombosis
Logistic	0.90 (0.90–0.90)	0.89 (0.89–0.89)	0.79 (0.79–0.79)	0.71 (0.71–0.71)
*l*1‐regularized logistic regression	0.90 (0.90–0.90)	0.90 (0.89–0.90)	0.80 (0.79–0.80)	0.73 (0.72–0.73)
k‐nearest neighbor classifier	0.89 (0.89–0.89)	0.88 (0.88–0.88)	0.78 (0.78–0.78)	0.71 (0.71–0.72)
Gradient boosting classifier	0.90 (0.90–0.90)	0.90 (0.90–0.90)	0.80 (0.80–0.80)	0.73 (0.72–0.73)

Data are shown as area under the curve (95% CI).

Furthermore, calibration plots indicated good calibration of all the classifiers for all 4 outcome scores in the validation set (Figure S1). Best Brier scores were achieved when predicting early mortality (range, 0.109–0.118) and intracranial mass effect (range, 0.113–0.132). Slightly higher scores were obtained for intracerebral hemorrhage (range, 0.165–0.173) and DVT (range, 0.191–0.213).

**Figure 1:**
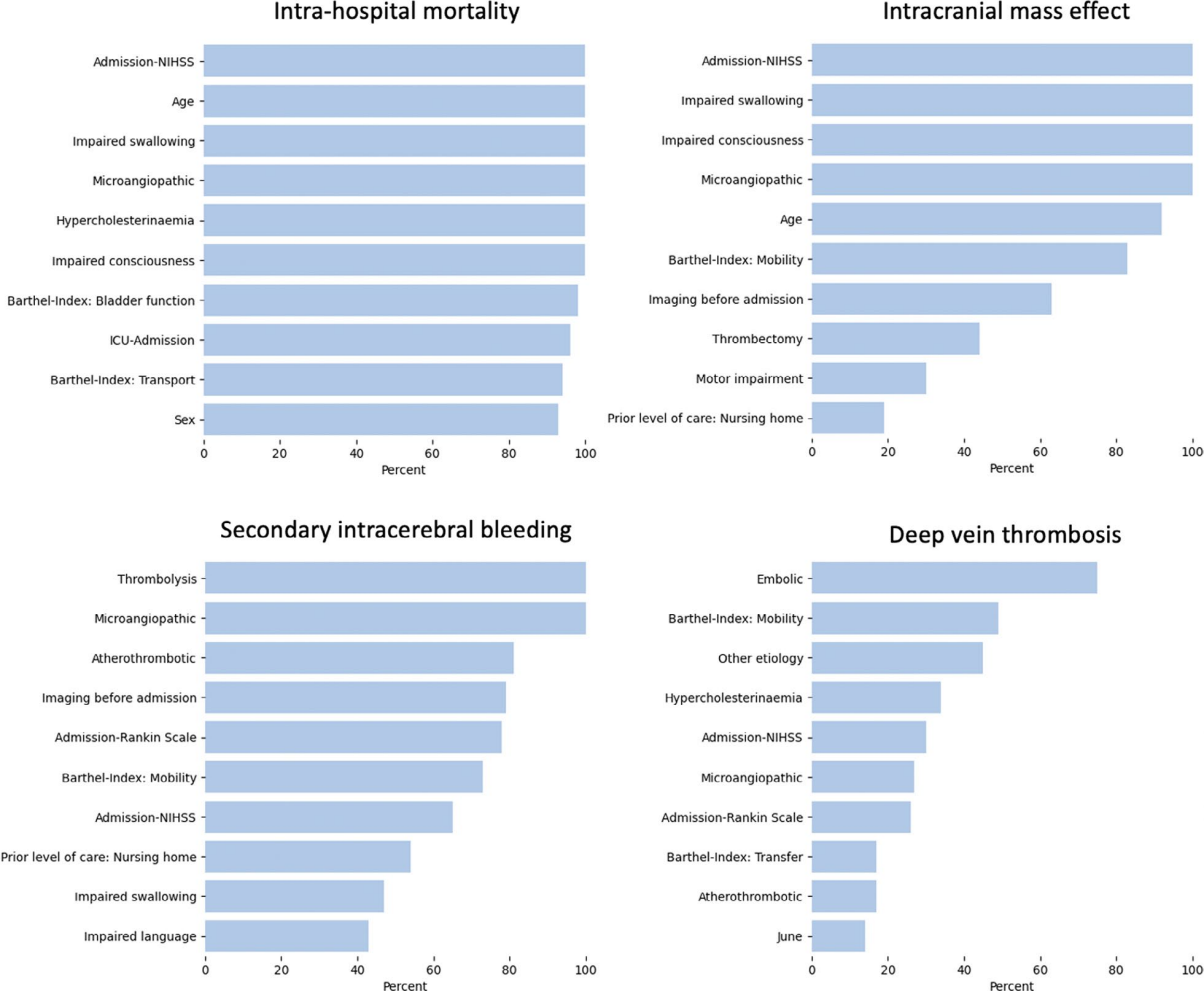
The 10 most frequently selected variables in the backward stepwise logistic regression models. After each initial downsampling step, we performed backward stepwise variable selection, that is, we only kept those input variables in the model that were significantly associated with the outcome. Because we repeated the downsampling step 100 times, an input variable could have, at maximum, been selected 100 times, or in 100% of the cases (x axis). Altogether, a variable may be considered more important in the prediction of a specific outcome, the more often it is selected. In case of secondary intracerebral hemorrhage, thrombolysis and microangiopathic stroke etiology were, for example, selected in all 100 downsampling scenarios and may thus possess the highest predictive capacity. Atherothrombotic stroke etiology and imaging before admission were selected in ≈80% of the downsampling scenarios and hence did not contribute to prediction models in ≈20% of the cases, indicating a less consistent predictive capacity. Of note, we here only measured the overall relevance, yet not the direction of the association. Each variable could thus have had a positive or negative effect on the outcome. In a second step, we retrained logistic models with the 10 most stables input variables in 100 further downsampled scenarios to compute odds ratios informing about the directionality of effects (Table S11). Tables S7 through S10 furthermore present the group averages for patients with and without a specific outcome. Because the outcome deep vein thrombosis could not be predicted as well as the other outcomes, the relevance of input variables was not as certain either, which may explain the lower overall percentages for selected variables. ICU indicates intensive care unit; NIHSS, National Institutes of Health Stroke Scale.

**Figure 2:**
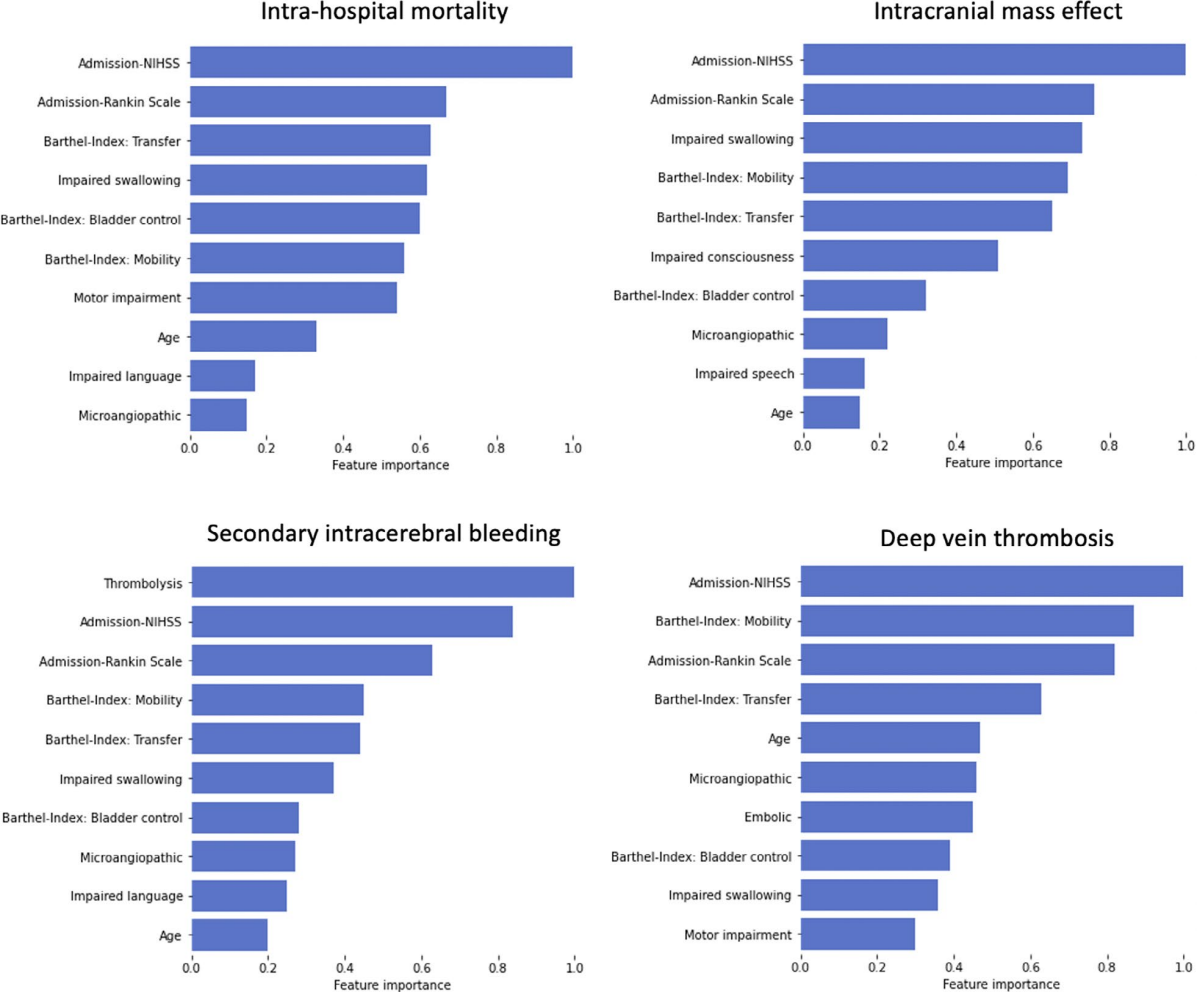
Feature importance for the 10 most important input variables for each of the 4 outcomes based on the gradient boosting classifier models. Feature importance, a measure inherent to tree‐based algorithms, is higher the more a variable contributes to the prediction of a specific outcome. Accordingly, the NIHSS score on admission was the most important variable in prediction of in‐hospital mortality, increased intracranial pressure, and deep vein thrombosis, whereas administration of thrombolytic therapy was the most telling variable in the prediction of an intracerebral hemorrhage. Individual feature importance has been normalized by the top‐ranked input variable and therefore range from 0 to 1. NIHSS indicates National Institutes of Health Stroke Scale.

The most relevant input variables of each outcome’s backward stepwise regression model are presented in Figure 1. Because we repeated the backward stepwise selection after each of the 100 downsampling steps in total, an input variable could have, at maximum, been selected 100 times, or in 100% of the cases. The more often a variable was selected, the more stably important it may be for outcome prediction. The input variables with the highest feature importance in the gradient boosting models are shown in Figure 2. Altogether, NIHSS upon admission was the input variable most frequently ranked first for both the backward stepwise regression models and the gradient boosting models. The median of the admission stroke severity was generally several points higher in the groups of patients who experienced a severe complication than in the control group (eg, in‐hospital mortality NIHSS: patients who died during the hospital stay, 17 [IQR, 11]; patients who survived, 4 [IQR, 5]; cf. Tables S7 through S10), which indicates a likely positive association between stroke severity and the adverse outcomes. This notion is reinforced by an odds ratio >1 in all 4 logistic regression models (cf. Table S11). For intrahospital mortality and intracranial mass effect, impaired swallowing and consciousness at admission as well as age had high overall rankings. The groups of patients who died during the hospital stay or were diagnosed with an intracranial mass effect had substantially higher percentages of impaired swallowing and impaired consciousness (eg, in‐hospital mortality and impaired swallowing: patients who died, 75.4%; patients who survived, 19.6%). In the case of in‐hospital mortality, patients who died were on average 8.5 years older than patients who survived; in the case of intracranial mass effect, this age difference was less pronounced. Odds ratios for these input variables were consistently >1, with a maximum of 2.75 (95% CI, 2.69–2.81) for impaired swallowing function in the prediction of intracranial mass effect. The uptake of intravenous thrombolysis was the most relevant input feature for the prediction of secondary intracerebral hemorrhages. Thrombolysis was ≈3 times more frequent in patients with secondary intracerebral hemorrhages than in patients without (48.1% versus 16.9%) and had an odds ratio of 3.49 (95% CI, 3.44–3.55). Of note, some high‐ranked input variables, such as microangiopathic etiology, were generally less frequent in the groups of patients with an adverse outcome, indicating negative associations to the adverse outcome. The exhaustive list of odds ratios and group averages for all the input variables stated in Figures 1 and 2 that inform about the likely directionality of effect can be found in Tables S7 through S11. Prediction results based on the left‐out test set for the 2016 development data set were very similar to the 2017 validation set results and can be found in Table S12.

## Discussion

We developed and validated prediction models for mortality and 3 further key severe complications after acute ischemic stroke. In‐hospital mortality as well as intracranial mass effects could be predicted with high accuracy and AUCs of 0.9. Prediction performances of intracerebral hemorrhage as well as DVT were capped at slightly lower levels, achieving AUCs of 0.80 and 0.73, respectively. Herewith we consistently achieved prediction performances beyond those reported in the literature.

### Early Mortality

Various studies aimed at constructing robust predictions models for mortality at varying time points after stroke. Frequently, these studies were also based on stroke registry data. Considered time points ranged from the early acute phase (<7 days)^4^ and in‐hospital mortality^19^, ^20^ to the subacute (eg, 30 days) and chronic phase 1 year after stroke.^21^, ^22^ In a recent study, Gattringer et al^4^ developed the predicting early mortality of acute ischemic stroke score for mortality within the first week after stroke, relying on Austrian stroke registry data of 77 653 patients with stroke. These data were pooled from the years 2006 to 2016. They achieved an AUC of 0.88 (95% CI, 0.86–0.91) in the temporal validation data set that considered data from patients with stroke in 2017. In view of our validation cohort AUC of 0.90 (95% CI, 0.90–0.90), we thus present a slighter better prediction performance that is, however, still included in the upper end of the 95% CI in Gattringer et al.^4^

Moreover, our results compare favorably with those obtained in the largest study in the field that considered data from 274 988 patients with ischemic stroke. These patients originated from 1036 hospitals participating in the American Get With the Guidelines Stroke Program between 2001 and 2007.^20^ The authors focused on in‐hospital mortality—thus an outcome comparable with ours—and established a validation data set prediction performance AUC of 0.72. Although this performance was obtained without any information on stroke severity, they augmented this performance to an AUC of 0.85 in a subsample of 109 187 patients who had admission NIHSS scores readily available. Interestingly, they reported an AUC of 0.83 for a model considering the NIHSS score only, which demonstrates the high relevance of initial stroke severity in the prediction of mortality.

All of these studies used slightly different clinical variables compared with those in our study. In most cases, these included age, some measure of stroke severity, and information on prestroke health status (eg, information on prior stroke; comorbidities, such as diabetes, preexisting heart disease). Extracted features of our best performing model, the gradient boosting classifier, also comprised measures of NIHSS‐derived stroke severity and Rankin Scale–based degree of disability. In addition, our model singled out impaired swallowing and several Barthel Index items, such as mobility and bladder control, as relevant predictive features.

### Intracranial Mass Effects

Numerous previous studies have focused on predicting mortality attributed to cerebral edema or cerebral edema with mass effects,^5^, ^23^ whereas our aim here was to predict the presence or absence of intracranial mass effects. A recent study more comparable with ours recruited 572 patients with ischemic stroke and described a prediction algorithm for cerebral edema with mass effect as detected on follow‐up scans 3 days after the acute event.^24^ Cerebral edema were observed in a fourth of the patients and could be predicted with an AUC of 0.78 (in‐sample estimate). Key predictors were total anterior circulation syndrome, hyperdense appearance of middle cerebral artery, closed eyes, vomiting (all positively associated), lacunar cerebral syndrome, and white matter lesions (negatively associated).

We observed a substantially higher prediction performance in our study as we achieved an AUC of 0.90 despite a simpler nature of our input variables. The most important predictors of our best performing model resembled those for the prediction of mortality and included the NIHSS, Rankin Scale, and Barthel Index items at admission as well as information on impairments of swallowing, consciousness, and stroke etiology (microangiopathic).

Our estimate was obtained in an independent validation data set, whereas Muscari et al^24^ reported in‐sample estimates that might be affected by overfitting. The study by Muscari et al and our study differed in their inclusion and exclusion criteria: Muscari et al excluded patients with visible edema at admission, which was not possible in the current study. Information on the exact time point of edema diagnosis was not available. Furthermore, the incidence of intracerebral edemas differed notably: 27.6% versus 1.7%. This difference might be partly, but likely not fully, explained by a more stringent and complete imaging follow‐up in Muscari et al and varying definitions of cerebral edema. It seems likely that their substantially smaller sample included patients who were more severely affected than would be observed in a more general sample as our stroke registry.

### Secondary Intracerebral Hemorrhage

Secondary intracerebral hemorrhage after initial ischemic stroke was previously mainly predicted in samples of patients who had received intravenous thrombolysis before study inclusion. The 2 most recent external validation studies report AUCs between 0.56 and 0.76 for various published intracerebral hemorrhage prediction algorithms.^25^, ^26^ Although Strbian et al^25^ declared a score abbreviated to SEDAN^6^ (SEDAN: baseline blood Sugar, Early infarct signs, [hyper] Dense cerebral artery sign on admission computed tomography scan, Age, NIH Stroke Scale on admission) as the best model (AUC=0.70), Asuzu et al^26^ instead reported best prediction performances for the DRAGON^27^ ([hyper] Dense cerebral artery sign/early infarct signs on admission CT scan, prestroke modified Rankin Scale score, Age, Glucose level at baseline, Onset‐to‐treatment time, NIH Stroke Scale on admission) score with an AUC of 0.73. Varying validation set sizes—3012 patients in the case of Strbian et al and 210 in Asuzu et al—may partially explain these differences. Furthermore, the DRAGON score was not tested in Strbian et al.

In contrast to these studies, we did not reduce our sample to only those patients who received intravenous thrombolysis. Instead, we predicted the occurrence of symptomatic secondary intracerebral hemorrhages in an unselected sample of patients with ischemic stroke. We considered this complementary approach worthwhile given that in our sample less than half of the patients with stroke experiencing a symptomatic cerebral hemorrhage had actually received a thrombolytic treatment (46.8% of 2576 patients with intracerebral hemorrhage). In this way, we also tried to prevent potential sample biases as, conceivably, multiple factors lead to the decision of intravenous thrombolysis in the first place.

Altogether, our results are promising because we could predict secondary intracerebral hemorrhages with an AUC of 0.80—higher than those reported in the literature. Furthermore, we only used routinely obtained clinical variables, whereas both the SEDAN as well as the DRAGON score build on more elaborated information. In addition to age and stroke severity, they consider blood glucose levels and imaging‐derived information on early infarct signs, such as a hyperdense cerebral artery sign, in the admission scans. Unsurprisingly, the administration of intravenous thrombolytic therapy was the input variable with the highest importance in predicting secondary intracerebral hemorrhages. Measures of stroke severity and stroke etiology were further important input features. Their importance in predicting secondary intracerebral hemorrhages may explain why prediction performance remained high, almost unchanged at an AUC of 0.79, when restricting the analyses to only those patients who did not receive any intravenous thrombolysis.

### Deep Vein Thrombosis

Especially in the presence of limb paralysis and immobility, DVT is a relevant outcome after stroke that can have severe consequences as it can cause a potentially fatal pulmonary embolism.^2^ Early mobilization, sufficient hydration, and also prophylactic anticoagulation can be seen as examples of effective preventive strategies.^28^, ^29^ Previous prediction modeling studies for DVT have resulted in rather mediocre prediction performances, importantly, despite comparatively comprehensive sets of clinical input features. Dennis et al^7^ used data from 2664 patients with stroke who were immobile originating from the CLOTS (Clots in Legs or Stockings After Stroke) trial to build a classifier of DVT occurrence. Forward and backward input variable selection processes led to the selection of the following variables as the most discriminative: dependence before stroke, unable to lift arms off bed, history of DVT/pulmonary embolism, and diabetes. The AUC, however, was only 0.57 in the validation data set. Smaller studies, recruiting 671 and 862 patients, reported slightly higher prediction performances with AUCs of 0.65 and 0.70, respectively.^30^, ^31^ In part, these scores also relied on more elaborate input variables, such as information on obesity, active cancer, or the level of low‐density lipoprotein. Because our highest validation data set prediction performance showed an AUC of 0.73, it was found to be higher than previous estimates—despite the simple nature of our input variables. However, clinical utility may still be limited in view of the absolute AUC value. An important difference between studies can once again be seen in markedly differing outcome proportions: only 0.4% of patients in our data set had a documented event of DVT, whereas signs of DVT were detected in 10.9% of the patients in Dennis et al,^7^ 12.4% of the patients in Liu et al,^31^ and 22.1% of the patients in Li et al.^30^ These differences may be partly explained by varying inclusion criteria—several of the previous studies focused on patients with an increased baseline risk, for example, attributed to severe hemiparesis. In addition, all of the aforementioned studies applied ultrasonography to screen for DVT. Currently, there is no such general ultrasonography screening established for unselected patient collectives, such as the collective represented in our stroke registry. As a result, the number of undetected cases may be high.

### General Considerations and Potential Outreach

In summary, we present evidence of augmented performance in predicting intrahospital mortality and 3 further important severe adverse outcomes after acute ischemic stroke. In contrast to the majority of previous studies, which primarily relied on logistic regression, we provide a comprehensive overview of the performance of multiple statistical learning approaches.^32^ We opted for approaches representing various different learning architectures. Some of them, the k‐nearest neighbor and gradient boosting classifiers, can automatically extract nonlinear effects. In the case of logistic regression, this is only possible if these effects are inserted manually and thus intentionally, likely relying on preexisting notions, often in the form of expert knowledge. However, we could, at best, detect marginal improvements through the application of advanced statistical learning approaches: gradient boosting outperformed logistic regression in all 4 cases. Nonetheless, AUC estimates differed by an amount of a maximum of 0.02 only. This finding of no substantial superiority of more advanced machine learning–like, model‐building strategies is well in line with several previous reports.^33^, ^34^ For instance, Evangelia et al^33^ reviewed 71 clinical prediction studies that compared logistic regression to machine‐learning approaches and could not detect any generally added benefit.

Although we did not observe a substantial improvement by advanced learning algorithms in comparison with logistic regression, our models still compared favorably with previously reported prediction models. Importantly, we achieved this performance despite generally simple input variables that were acquired in clinical routine. The observed increase in prediction performance may be explained by a larger data set size in comparison with earlier studies: our models had access to more incident cases to learn classification rules from. We also took into account a great variety of input variables and did not reduce our model to very few select variables. Moreover, increased data homogeneity may have been instrumental, as data were exclusively obtained in 2016 and 2017. Acute stroke treatments have experienced monumental changes, ranging from the introduction of stroke unit care in the 1990s to thrombolysis in the 2000s, more advanced imaging, extension of pertinent time windows, and nationwide thrombectomy in the 2010s.^8^ In addition, the population of patients with stroke as a whole has experienced substantial alterations, primarily attributed to aging effects. All of these changes in the characteristics and care of patients with stroke have potential effects on functional outcomes and complications after stroke as well as on how these end points can be predicted.

Thus, any prediction model and its implementation should match these dynamics. We concentrated on a short, homogeneous time window from 2016 to 2017 that already reflects the most fundamental changes in acute stroke management, such as thrombolysis and thrombectomy. Our models would, however, have to be updated regularly to adopt to gradually changing environments in parallel. Such an update would be a feasible plan in the case of German stroke registry data, as these kinds of data are forwarded to a central storage in short time intervals.

A strength of our study can be seen in the large number of individual patients who originated from 155 hospitals, which underlines the chances of successful generalization. As our stroke registry data are anonymized and already stored centrally, its use does not interfere with data privacy concerns or often experienced barriers to shared data.

Altogether, our findings may argue for the use of routinely acquired clinical data to build outcome prediction models with the goal to stratify patients according to their risk profiles—the prediction models used here were all able to generate probability estimates of an outcome. Although it may not be feasible to apply preventive measures for unselected patient collectives, such a risk stratification could then allow for more tailored approaches. Preventive actions could then only be considered for those patients with the highest risks and potentially help to alleviate the detrimental effects of all of these complications—in the short and long term. Concretely, we could, for example, identify a subgroup of patients with stroke with a high DVT risk, say >80%, and administer venous Doppler of the legs before hospital discharge specifically to this subgroup. At best, this personalized diagnostic scheme would help uncover relevant cases of DVT early while being economical and not overstraining clinical resources. By these means, this risk stratification approach is conceptually different from prognostication: we assume that a specific outcome is still malleable in the first case, for example, by treatments, whereas it is fixed in the second case.

A limitation of our study may be the decreased level of model transparency. Because we strived for the highest possible prediction accuracy, we accepted a decrease in interpretability.^35^, ^36^, ^37^ We could, however, generate rankings of most stably selected and important input variables (Figures 1 and 2). We then gained some insights on the likely directionality of effects for these relevant input variables via comparing group averages for patients with and without a specific outcome. Further options may be seen in general post hoc explanation methods, such as LIME^38^ (locally interpretable model‐agnostic explanations) and SHAP^39^ (Shapley Additive Explanations), which can be employed to any model to generate explanations of the model’s output.

A further limitation of our work may be that we did not test any deep‐learning approaches. Typical studies in areas where deep learning excels, for example, in image or language processing, consider training examples in the millions.^40^ In addition, deep learning has been shown to be particularly beneficial if complex interactions are present and exploitable.^41^, ^42^ Consequently, a significant increase in performance appears rather unlikely given the limited data set size and lack of signs of nonlinear or interaction effects. Furthermore, although the usage of routinely acquired, basic clinical data may increase the feasibility of application in clinical settings, it may also be considered a limitation of our study: conceivably, information on more elaborated laboratory values^43^ or advanced neuroimaging findings, such as vessel occlusions or infarct size or locations,^44^, ^45^ which we did not have access to in our stroke registry, can enhance prediction performances beyond those observed here. In addition, future studies may consider predicting continuous instead of our dichotomous outcome measures to further increase clinical utility. Most stroke registry data are acquired by experienced physicians, the data itself are continuously curated and carefully quality controlled at the Institute of Epidemiology, University of Muenster. Nonetheless, our stroke registry data represent observational data and feature some missing data (up to 7%, as demonstrated here), which may warrant future (prospective) studies to ensure a reliable generalization of our models to completely new patient data. Lastly, differences in sample characteristics and rates of outcomes may hamper direct comparisons between studies. Given that these differences were, for example, very pronounced in the case of DVT, future studies could examine whether our improved prediction performance was primarily attributed to predicting more severe and clinically particularly relevant cases of DVT that were documented by the clinical team.

In future work, we plan to extend prediction scenarios to intracerebral hemorrhage after intravenous thrombolysis, mortality after intracranial mass effects, and infectious events, such as pneumonia, after acute ischemic stroke. Our code and current versions of our trained models, which can be used to generate outcome predictions for new patients, is openly available on GitHub. We will furthermore aim to update models regularly to match training sets to current stroke populations as closely as possible. Lastly, it will be important to validate models in temporally as well as spatially independent data sets.

## Conclusions

Using data from 152 710 patients included in the stroke registry, we presented validated prediction models for 4 key adverse outcomes after acute ischemic stroke. We achieved high prediction accuracies for in‐hospital mortality as well as intracranial mass effects with AUCs of 0.90. Intracerebral hemorrhage as well as DVT were predicted with AUCs of 0.80 and 0.73, respectively. Herewith, our prediction models consistently performed favorably when compared with previously established models. The NIHSS‐defined stroke severity upon admission was the most predictive input variable for all outcomes. In view of their excellent AUCs, prediction models for in‐hospital mortality as well as intracranial mass effects could be of clinical importance and augment the clinical decision‐making process.

## Sources of Funding

Dr Bonkhoff is supported by a Massachusetts General Hospital Executive Committee on Research Fund for Medical Discovery Clinical Research Fellowship Award. Dr Grefkes is in part funded by the Deutsche Forschungsgemeinschaft (German Research Foundation; project 431549029) and SFB 1451 projects B05 and C05. Dr Rost is supported in part by the National Institutes of Health–National Institute of Neurological Disorders and Stroke (R01NS082285, R01NS086905, U19NS115388).

## Disclosures

Dr Rost has received compensation as scientific advisory consultant from Omniox, Sanofi Genzyme, and AbbVie Inc. The remaining authors have no disclosures to report.

## Supporting information

Data S1–S3
Tables S1–S12
Figure S1
References 46, 47
