# Supplementary material for: Development and Validation of Prediction Models for Severe Complications After Acute Ischemic Stroke: A Study Based on the Stroke Registry of Northwestern Germany
Source: J Am Heart Assoc. 2022 Mar 5;11(6):e023175. doi: 10.1161/JAHA.121.023175 (PMC9075320; doi:10.1161/JAHA.121.023175)
Supplement: Supplementary file 1 — Data S1–S3 Tables S1–S12 Figure S1 References 46, 47 [file JAH3-11-e023175-s001.pdf]

SUPPLEMENTAL MATERIAL

Data S1. Data collection tool 2016 – English translation

We here present an exhaustive list of obtained items. We only considered those variables for our prediction tool that were readily available upon admission and continuously documented for the years 2016 and 2017. The original German version can be accessed online: https://www.medizin.uni-muenster.de/qsnwd/downloads.html ("Spezifikationen").

Center

Date of admission

Date of discharge

Year of birth

Patient's provision available – yes/no

Sex – Male/Female

Time between symptom onset and admission – <1h, 1-2h, 2-3h, 3-3.5h, 3.5h-4h, 4-6h, 6-24h, 24-48h, >48h, unknown

Duration of symptoms – <1h, 1-24h, >24h

Transport to Hospital – private, emergency doctor/helicopter with emergency doctor, ambulance (without emergency doctor), other

Admission initiated by – self, emergency doctor, doctor in private practice, external hospital, internally in hospital

Barthel-Index upon admission and at discharge - Bladder control, Transition Bed-Chair, Mobility

Initial admission on – normal ward, intensive care, stroke unit

Stay on Stroke Unit – yes/no

Days in Stroke Unit

Symptoms upon admission:

Motor (Arm/Hand and/or Leg/Foot) – yes/no

Speech – yes/no

Language – yes/no

Swallowing – yes/no

Consciousness – awake, somnolent, comatose

Comorbidity:

Diabetes mellitus – yes/no

Hypertension – yes/no

Previous myocardial infarction – yes/no

Previous stroke – yes/no

Hypercholesterinaemia – yes/no

Atrial fibrillation – yes, known, yes, but newly diagnosed, no

ICD-10 classification

Etiology – no infarct, atherothrombotic, cardiogen-embolic, microangiopathic, other cause, unknown cause, competing causes

Diagnostics after event:

Long-term ECG (at least 24h) – yes/no

Swallowing test – yes/no

Neuroimaging: CCT – yes/no

Neuroimaging: MRI – yes/no

If Neuroimaging: New visible lesion – yes/no

Extracranial vascular diagnostics – no, yes in <48h, yes in >48h

Intracranial vascular diagnostics – no, yes in <48h, yes in >48h

If vascular diagnostics: Symptomatic ipsilesional stenosis of Ateria carotis interna – no, <50%, 50-70%, 70-99%, 100%, not investigated

Recommendation of revascularization – no, surgery documented, stenting documented, transfer for surgery, transfer for stenting, other

Therapy/Prophylaxis:

Antiplatelet agent <48h after symptom onset – yes/no

Antiplatelet agent at discharge – yes/no

Thromboprophylaxis – yes/no

Oxygen – yes/no

Antihypertensives – yes/no

Antidiabetics – yes/no

Statins – yes/no

Anticoagulation (e.g. Warfarin) – no, Vitamin K-Antagonists, new anticoagulants

Complications – no complications, urinary tract infection, pneumonia, re-stroke, increased intracranial pressure, intracerebral bleeding, thrombosis, other complication

Rehabilitative therapy:

Physio-/ Occupational therapy – no, yes (begin <2days after onset), yes (begin >2days after onset)

Speech therapy – no, yes (begin <2days after onset), yes (begin >2days after onset)

Mobilization – no, yes (begin <2days after onset), yes (begin >2days after onset)

Thrombolysis i.v. – yes/no

Thrombolysis i.a. – yes/no

Thrombectomy – yes/no

NIHSS upon admission

Time between admission and first neuroimage – no image, first image before admission, <0.5h, 0,5-1h, 1-3h, 3-6h, >6h

Time between admission and thrombolysis (i.v., i.a.) – no thrombolysis, <0.5, 0.5-1h, 1-2h, 2-3h, 3-4h, 4-6h, >6h
Planned rehabilitation after discharge from acute care hospital – no rehabilitation planned, neurological rehabilitation Phase B, neurological rehabilitation Phase C, neurological rehabilitation Phase D (inpatient), neurological rehabilitation Phase D (outpatient), geriatric rehabilitation, other rehabilitation
Discharge – regular discharge, discharge due to other reasons, transfer to other facility <2h after admission, transfer to other hospital, death, discharge to rehabilitation, discharge to nursing home, internal transfer

Data S2. Complete case analyses

We performed complete case analyses and thus excluded subjects with missing information for any of the considered variables. This led to the exclusion of 1,270 out of 76,019 patients for the year 2016 (1.7%) and the exclusion of 5,378 patients out of 76,691 patients (7.0%) for the year 2017. The exclusion of more patients in 2017 than 2016 (7% vs. 1.7%) was primarily due to an increased frequency of missing information on "Swallowing impairments at admission" (3.0% in 2017 vs. 0.4% in 2016), "Speech impairments at admission" (2.1% vs. 0.2%), "Language impairments at admission" (1.4% vs. 0.1%) and the comorbidities hypercholesterinaemia (1.8% vs. 0.4%) and prior myocardial infarct (1.7% vs. 0.2%). This increase between 2016 and 2017 may be due to the fact that it was possible to record these items as "present/absent/not possible to determine" in 2017, but only as "present/absent" in 2016.

The comparison of included and excluded patients based on the complete case criterion can be found in **Tables S2-S3**. The groups of excluded patients in 2016 and 2017 were slightly older, comprised more female patients and had a slightly higher median Rankin Score in 2017 than the one of included patients. Included and excluded subjects in the 2016 derivation cohort, used for model training, differed in only very small absolute amounts. Differences were slightly more pronounced in the 2017 validation cohort. However, importantly, differences in key characteristics (age, sex, Rankin Scale) and rates of the four adverse outcomes of *included* subjects in 2016 and 2017 were very small in magnitude. This later aspect is reassuring with respect to the suitability of the 2017 cohort as validation cohort.

Data S3. Details on our prediction pipeline

Downsampling

The downsampling step was motivated by pronounced class imbalances in case of all four of our adverse outcomes (rates: 5% mortality, 1.7% ICP, 1.8% ICH and 0.4% DVT). Downsampling the majority class, as implemented in our study, has been shown to improve classifier performance on minority class cases, i.e., those patients that are usually of interest.[46]

Variable Normalization

As some of the used approaches, i.e., the *l*1-regularized logistic regression and k-nearest neighbor classifier, are sensitive to variable scales, we normalized input variables by subtracting the sample mean and dividing by the sample standard deviation in the training set and applied the same normalization to the test set.

Feature importance

The feature importance computed for the gradient boosting classifier quantifies how much each variable improves the prediction performance based on the increase in the Gini index-derived purity. The Gini index itself describes the total variance across various classes; a low value indicates that each node contains only observations from a single class.[47]

Table S1. Stroke sample characteristics (continuation).

		**2016 and 2017**
**Months**	**Jan**	12,464 (8.5)
	**Feb**	11,603 (7.9)
	**Mar**	12,541 (8.6)
	**April**	11,924 (8.2)
	**May**	12,596 (8.6)
	**June**	12,019 (8.4)
	**July**	12,275 (8.4)
	**August**	12,345 (8.5)
	**September**	11,847 (8.1)
	**October**	12,043 (8.2)
	**November**	12,110 (8.3)
	**December**	11,530 (7.7)

Table S2. Key characteristics of included and excluded subjects in the year 2016.

	Included subjects (N=74,749)	Excluded subjects (N=1,270)	Statistical comparison (two-sided t-test or Fisher's exact test as appropriate)
**Age (in years)**	72.7 (13.1)	73.3 (12.7)	p=0.08
**Sex (female)**	47.6%	48.1%	p=0.77
**Rankin Scale upon admission**	3 (2)	3 (2)	p=0.01

Table S3. Key characteristics of included and excluded subjects in the year 2017.

	Included subjects (N=71,313)	Excluded subjects (N=5,378)	Statistical comparison (two-sided t-test or Fisher's exact test as appropriate)
**Age (in years)**	72.7 (13.0)	74.9 (12.8)	p<0.01
**Sex (female)**	47.1%	52.7%	p<0.01
**Rankin Scale upon admission**	3 (2)	4 (2)	p<0.01

Table S4. Key characteristics and frequencies of adverse outcomes of included subjects in 2016 and 2017.

	**Derivation cohort (2016)** (N=74,749)	**Validation cohort (2017)** (N=71,313)
**Age (in years)**	72.7 (13.1)	72.7 (13.0)
**Sex (females)**	35,616 (47.7)	33,618 (47.1)
**Rankin Scale upon admission**	3 (3)	3 (2)
**In-hospital mortality**	4,046 (5.4)	3,637 (5.1)
**Increased intracranial pressure**	1,339 (1.8)	1,072 (1.5)
**Secondary intracerebral hemorrhage**	1,361 (1.8)	1,219 (1.7)
**Deep vein thrombosis**	337 (0.5)	269 (0.4)

Table S5. Model building approaches and their respective hyperparameter settings entered in the grid search.

**Approach**	**Hyperparameter**	**Description of hyperparameter**	**Subset of values assessed in the grid search**
*l*1-regularized	'C'	'C': inverse of regularization strength. Smaller values imply a stronger regularization.	'C' = 0.01, 0.1, 1, 10, 100, 1000

logistic regression			
k-nearest neighbor classifier	'n_neighbors’	'n_neighbors’: number of nearest neighbors to respect for majority vote (i.e., a new data point is classified into the same group than the majority of considered neighbors)	'n_neighbors’ = 1, 5, 10, 50
Gradient boosting classifier	‘n_estimators’<br>‘max_depth’<br>‘loss’	‘n_estimators’: the number of boosting stages to conduct<br>‘max_depth’: maximum depth of individual classifiers<br>‘loss’: loss function that is optimized	‘n_estimators’ = 100, 300, 500<br>‘max_depth’ = 1, 3, 5<br>‘loss’ = 'deviance', 'exponential'

Table S6. Validation dataset AUCs for the prediction of secondary intracerebral hemorrhage in the sample of stroke patients that did not receive any thrombolytic therapy.

**Classifier**	**Secondary intracerebral hemorrhage**
**Logistic**	0.78 (0.78-0.78)
***l*1-regularized logistic regression**	0.79 (0.79-0.79)
**kNN**	0.77 (0.77-0.77)
**GBC**	0.79 (0.79-0.79)

Table S7. Group averages for most stable and important input variables for in-hospital mortality in 2016.

	**Patients without in-hospital mortality**	**Patients with in-hospital mortality**
**Both logistic regression and gradient boosting**		
**Admission-NIHSS**	4 (5)	17 (11)
**Age**	72.2 (13.1)	80.7 (10.2)
**Impaired swallowing**	19.6%	75.4%
**Microangiopathic etiology**	20.9%	3.3%
**Barthel index: Bladder function (less than full score)**	30.9%	86.1%
**Barthel Index: Transfer (less than full score)**	58.5%	96.2%
**Only logistic regression**		
**Hypercholesterinaemia**	57.1%	43.3%
**Impaired consciousness**	6.2%	52.6%
**ICU-admission**	4.4%	22.9%
**Sex (female)**	47.0%	58.3%
**Only Gradient boosting**		
**Admission-Rankin Scale**	3 (5)	5 (1)
**Barthel Index: Mobility (less than full score)**	65.7%	97.3%

**Motor impairment**	64.7%	92.9%
**Impaired language**	29.5%	70.8%

Table S8. Group averages for most stable and important input variables for increased intracranial pressure in 2016.

	**Patients without ICP**	**Patients with ICP**
**Both logistic regression and gradient boosting**		
**Admission-NIHSS**	4 (6)	18 (11)
**Impaired swallowing**	21.5%	79.3%
**Impaired consciousness**	7.9%	55.0%
**Microangiopathic etiology**	20.3%	1.6%
**Age**	72.7 (13.0)	73.3 (14.5)
**Barthel Index: Mobility (less than full score)**	66.8%	96.2%
**Only logistic regression**		
**Imaging before admission**	9.5%	17.3%
**Thrombectomy**	7.0%	25.9%
**Motor impairment**	65.7%	92.0%
**Prior level of care: Nursing home**	9.1%	12.8%
**Only Gradient boosting**		
**Admission-Rankin Scale**	3 (2)	5 (0)
**Barthel Index: Transfer (less than full score)**	59.9%	94.7%
**Barthel Index: Bladder control (less than full score)**	33.0%	80.0%
**Impaired speech**	43.5%	77.6%

Table S9. Group averages for most stable and important input variables for secondary intracerebral hemorrhage in 2016.

	**Patients without ICB**	**Patients with ICB**
**Both logistic regression and gradient boosting**		
**Thrombolysis**	16.9%	48.1%
**Microangiopathic etiology**	20.2%	5.1%
**Admission-Rankin Scale**	3 (2)	4 (2)
**Barthel Index: Mobility (less than full score)**	67.0%	91.3%
**Admission-NIHSS**	4 (6)	12 (12)
**Impaired swallowing**	22.0%	55.9%
**Impaired language**	31.3%	59.1%
**Only logistic regression**		
**Atherothrombotic etiology**	23.3%	19.5%
**Imaging before admission**	9.5%	14.1%
**Prior level of care: Nursing home**	9.1%	11.9%
**Only Gradient boosting**		
**Barthel Index: Transfer (less than full score)**	60.0%	88.8%

**Barthel Index: Bladder control (less than full score)**	33.3%	65.8%
**Age**	72.6 (13.1)	75.4 (12.2)

Table S10. Group averages for most stable and important input variables for deep vein thrombosis in 2016.

	**Patients without DVT**	**Patients with DVT**
**Both logistic regression and gradient boosting**		
**Embolic etiology**	31.9%	51.9%
**Barthel Index: Mobility (less than full score)**	75.6%	82.5%
**Admission-NIHSS**	4 (6)	9 (12)
**Microangiopathic etiology**	20.0%	6.5%
**Admission-Rankin Scale**	3 (2)	4 (2)
**Barthel Index: Transfer (less than full score)**	76.0%	80.4%
**Only logistic regression**		
**Other etiology**	3.4%	7.4%
**Hypercholesterinaemia**	56.4%	44.8%
**Atherothrombotic etiology**	23.2%	15.7%
**June**	8.3%	11.6%
**Only Gradient boosting**		
**Age**	72.7 (13.1)	72.6 (13.1)
**Barthel Index: Bladder control (less than full score)**	33.8%	57.6%
**Impaired swallowing**	22.5%	45.4%
**Motor impairment**	66.1%	81.3%

Table S11. Odds ratios for the ten most stables input variables of all four severe adverse outcomes. Logistic regression models for all four outcomes were re-run in 100 downsampling scenarios, this time considering the most stably selected input variables (instead of backward stepwise selection).

	**Odds ratio (95% confidence intervals)**
**In-Hospital mortality**	
**Admission-NIHSS per point increase**	1.08 (1.08-1.08)
**Age per year increase**	1.05 (1.05-1.05)
**Impaired swallowing**	2.15 (2.13-2.17)
**Microangiopathic etiology**	0.33 (0.32-0.33)
**Hypercholesterinaemia**	0.67 (0.66-0.67)
**Impaired consciousness**	2.13 (2.10-2.16)
**Barthel index: Bladder function (higher score higher function)**	0.95 (0.95-0.95)
**ICU-Admission**	1.68 (1.64-1.71)
**Sex (female)**	0.76 (0.75-0.77)

**Barthel Index: Transfer (higher score higher function)**	0.93 (0.93-0.93)
**Increased intracranial pressure**	
**Admission-NIHSS per point increase**	1.08 (1.07-1.08)
**Impaired swallowing**	2.75 (2.69-2.81)
**Impaired consciousness**	2.03 (1.99-2.08)
**Microangiopathic etiology**	0.21 (0.20-0.21)
**Age per year increase**	0.98 (0.98-0.98)
**Barthel Index: Mobility (higher score higher function)**	0.90 (0.90-0.90)
**Imaging before admission**	1.64 (1.60-1.68)
**Thrombectomy**	1.53 (1.50-1.57)
**Motor impairment**	0.68 (0.67-0.69)
**Prior level of care: Nursing home**	0.73 (0.70-0.75)
**Secondary intracerebral hemorrhages**	
**Thrombolysis**	3.49 (3.44-3.55)
**Microangiopathic etiology**	0.36 (0.36-0.37)
**Atherothrombotic etiology**	0.62 (0.61-0.63)
**Imaging before admission**	1.69 (1.66-1.73)
**Admission-Rankin Scale per point increase**	1.22 (1.21-1.23)
**Barthel Index: Mobility (higher score higher function)**	0.95 (0.95-0.96)
**Admission-NIHSS per point increase**	1.04 (1.04-1.04)
**Prior level of care: Nursing home**	0.67 (0.66-0.68)
**Impaired swallowing**	1.41 (1.38-1.43)
**Impaired language**	1.30 (1.28-1.32)
**Deep vein thrombosis**	
**Embolic etiology**	1.58 (1.53-1.64)
**Barthel Index: Mobility (higher score higher function)**	0.96 (0.96-0.96)
**Other etiology**	2.26 (2.09-2.45)
**Hypercholesterinaemia**	0.64 (0.63-0.66)
**Admission-NIHSS per point increase**	1.03 (1.03-1.04)
**Microangiopathic etiology**	0.51 (0.49-0.53)
**Admission-Rankin Scale per point increase**	1.16 (1.15-1.18)
**Barthel Index: Transfer (higher score higher function)**	0.99 (0.98-0.99)
**Atherothrombotic etiology**	0.68 (0.66-0.71)
**June**	1.87 (1.79-10.96)

Table S12. Test AUCs (95%-CI) in the training data from 2016.

**Classifier**	**Early mortality**	**Increased intracranial pressure**	**Secondary intracerebral hemorrhage**	**Deep vein thrombosis**
**Logistic**	0.91 (0.91-0.91)	0.89 (0.89-0.89)	0.80 (0.80-0.81)	0.70 (0.69-0.71)
***l*1-regularized logistic regression**	0.91 (0.91-0.91)	0.89 (0.89-0.89)	0.80 (0.80-0.81)	0.71 (0.70-0.72)
**kNN**	0.90 (0.90-0.90)	0.88 (0.87-0.88)	0.79 (0.79-0.79)	0.71 (0.69-0.71)

**GBC**	0.90 (0.90-0.90)	0.89 (0.89-0.89)	0.81 (0.80-0.81)	0.71 (0.71-0.72)

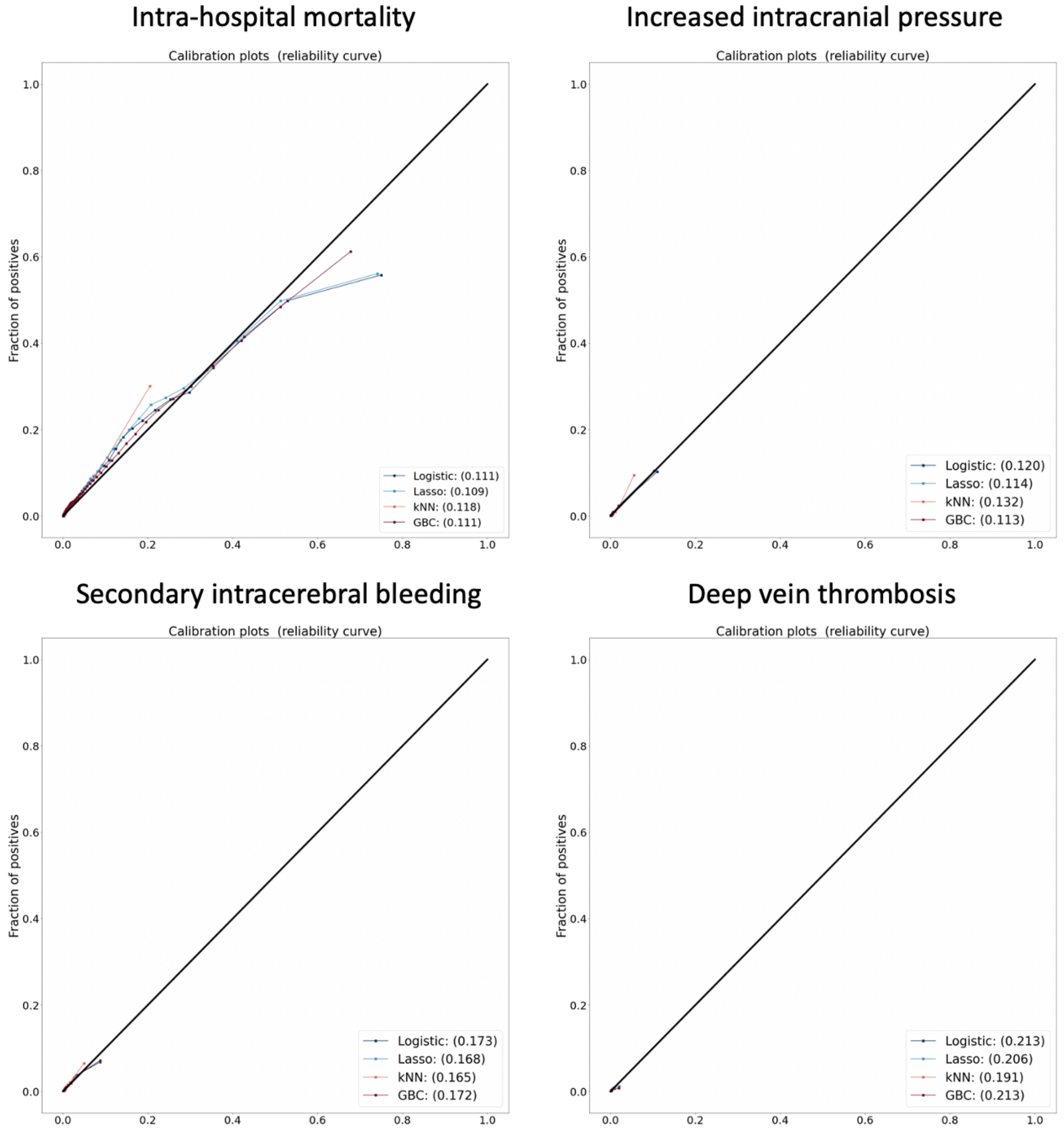

Figure S1. Calibration plots and Brier-Scores (c.f., figure legends) in the validation cohort. Calibration plots contrast the predicted probability of an outcome with the observed proportions. A model is considered well calibrated if those two measures correspond to one another closely. Brier scores measure the accuracy of probabilistic predictions, with lower values indicating a more favorable calibrations of predictions.
